# Formulation and assessment of biological properties of *garcinia indica* fruit extract mouthrinse as an adjunct to oral hygiene regimen: an *in vitro* analysis

**DOI:** 10.1590/1678-7757-2023-0291

**Published:** 2024-05-24

**Authors:** Shravya MACHERLA, Jothi VARGHESE, Usha Y NAYAK, Varalakshmi VELAGACHERLA, Richard LOBO, Viswanatha U, Vinayak KAMATH

**Affiliations:** 1 Manipal Academy of Higher Education Manipal College of Dental Sciences Department of Periodontology Manipal Karnataka India Manipal Academy of Higher Education, Manipal College of Dental Sciences, Department of Periodontology, Manipal, Karnataka, India.; 2 Manipal Academy of Higher Education Manipal College of Pharmaceutical Sciences Department of Pharmaceutics Manipal Karnataka India Manipal Academy of Higher Education, Manipal College of Pharmaceutical Sciences, Department of Pharmaceutics, Manipal, Karnataka, India.; 3 Manipal Academy of Higher Education Manipal College of Pharmaceutical Sciences Department of Pharmacognosy Manipal Karnataka India Manipal Academy of Higher Education, Manipal College of Pharmaceutical Sciences, Department of Pharmacognosy, Manipal, Karnataka, India.; 4 Sri Dharmasthala Manjunatheshwara Centre for Research in Ayurveda & Allied sciences Department of Biotechnology and Microbiology Udupi Karnataka India Sri Dharmasthala Manjunatheshwara Centre for Research in Ayurveda & Allied sciences, Department of Biotechnology and Microbiology, Kuthpady, Udupi, Karnataka, India.; 5 Goa Dental College and Hospital Department of Public Health Dentistry Bambolim Goa India Goa Dental College and Hospital, Department of Public Health Dentistry, Bambolim, Goa, India.

**Keywords:** Garcinia indica, Fruit extract, Mouthrinse, Turmeric mouthrinse, Gingivitis, Cytotoxicity, Staining, Substantivity

## Abstract

The prevalence of gingivitis is substantial within the general population, necessitating rigorous oral hygiene maintenance.

**Objective:**

This study assessed a *Garcinia indica* (GI) fruit extract-based mouthrinse, comparing it to a 0.1% turmeric mouthrinse and a 0.2% Chlorhexidine (CHX) mouthrinse. The evaluation encompassed substantivity, staining potential, antimicrobial efficacy and cytocompatibility.

**Methodology:**

The study employed 182 tooth sections. For antimicrobial analysis, 64 extracted human teeth coated with a polymicrobial biofilm were divided into four groups, each receiving an experimental mouthrinse or serving as a control group with distilled water. Microbial reduction was assessed through colony forming units (CFU). Substantivity was evaluated on 54 human tooth sections using a UV spectrophotometer, while staining potential was examined on 64 tooth sections. Cytocompatibility was tested using colorimetric assay to determine non-toxic levels of 0.2% GI fruit extract, 0.1% Turmeric, and 0.2% CHX.

**Results:**

Data were analysed with one-way ANOVA (α=0.05). Cell viability was highly significant (p<0.001) in the 0.2% GI group (64.1±0.29) compared to 0.1% Turmeric (40.2±0.34) and 0.2% CHX (10.95±1.40). For antimicrobial activity, both 0.2% GI (20.18±4.81) and 0.2% CHX (28.22±5.41) exhibited no significant difference (P>0.05) at end of 12 hours. However, 0.1% Turmeric showed minimal CFU reduction (P<0.001). Substantivity results at 360 minutes indicated statistically significant higher mean release rate in 0.1%Turmeric (12.47±5.84 ) when compared to 0.2% GI (5.02±3.04) and 0.2% CHX (4.13±2.25) (p<0.001). The overall discoloration changes (∆E) were more prominent in the 0.2% CHX group (18.65±8.3) compared to 0.2% GI (7.61±2.4) and 0.1% Turmeric (7.32±4.9) (P<0.001).

**Conclusion:**

This study supports 0.2% GI and 0.1% Turmeric mouth rinses as potential natural alternatives to chemical mouth rinses. These findings highlight viability of these natural supplements in oral healthcare.

## Introduction

Gingivitis, a globally renowned disease is defined as a non-specific inflammatory condition and is therefore a consequence of sustained plaque biofilm accumulation at and apical to the gingival margin.^1^ This inflammatory condition could result in redness of gingiva, bleeding on tooth brushing, halitosis etc. Regular tooth brushing is a widely adopted oral care practice, for mechanical removal of dental plaque. However, with the limited entry of the bristles into the inaccessible areas of the teeth, the necessity for chemical plaque control management has been suggested. This may be supplemented in the form of mouthwashes, gels, sprays etc.^2^ Mouthwashes, historically used for their antimicrobial properties and refreshing effects, offer benefits to the oral tissues owing to its sustained release, making them advantageous for oral hygiene.^3 , 4^ Since 1960’s, Chlorhexidine (CHX) has been widely regarded as the preferred choice among the realm of antimicrobial and anti-plaque agents.^5^ This cationic broad-spectrum anti microbial agent acts by binding to the tooth surface, impeding pellicle formation and disrupting the outer bacterial material, thereby inhibiting bacterial cell wall adsorption and mature plaque adherence.^6 , 7^ It is primarily recommended as supplementary to mechanical plaque control particularly in specific clinical conditions which include oral malodour, post-surgery, during fixed orthodontic therapy, individuals with intellectual and physical disabilities etc.^8^ Nevertheless, its prolonged use can lead to certain reversible side effects, including alteration in food taste, irritation of the oral mucosa^9 , 10^and the development of discoloration on oral soft tissues and aesthetic restoration. These unpleasant effects have the potential to negatively affect patient’s willingness to adhere to treatment.^8 , 11^ Literature has also revealed its cytotoxic effects on human gingival fibroblasts,^12^ periodontal ligament cells,^13^ and osteoblastic cells.^14^To minimize the adverse effects of chemical agents, the exploration of natural plant-based alternatives have gained interest. Several randomized controlled trials have demonstrated the effectiveness of varied herbal mouth rinses in combating gingival and periodontal diseases.^15^Among these, Turmeric, a rhizome of *Curcuma longa* , a herb recognized for its extensive medicinal properties have been found effective for dental diseases.^16^ Furthermore scientific literature has documented turmeric mouth rinse to be equally effective as the standard CHX mouth rinse in treating gingivitis.^17 , 18 , 19^Thus, it can be integrated as a valuable adjunct to mechanical plaque control techniques. Another natural plant with diverse medicinal properties is *Garcinia indica* (GI). This plant from the mangosteen family is renowned for its culinary, cosmetic and pharmaceutical uses. Traditionally, it was used as a remedy for systemic infections, due to its varied therapeutic benefits which include anti-inflammatory, anti-oxidant, anti-bacterial, cardioprotective etc.^20 , 21^ The leaves of GI and it’s fruit rind extracts, both in aqueous and menthol mediums, exhibit antibacterial effect.^22^ Phytochemical analysis of the fruit rind revealed the presence of Garcinol, hydroxycitric acid, and anthocyanin which were responsible for its remedial effects like anti-inflammatory, cell-scavenging and for reducing edema.^20^ A recent study investigated the antibacterial effects of different herbal extracts against oral bacteria. The authors found GI extract displayed the highest antimicrobial efficacy compared to all other tested herbs.^23^ In addition to their antibacterial properties, mouth rinses should demonstrate substantivity, be non-toxic, and should not discolour teeth. Majority of the earlier research on herbal mouth rinses have emphasized primarily on its antibacterial activity. Literature related to substantivity, discoloration potential and cytotoxicity of mouthrinses are limited. Therefore, the present study aimed to assess the substantivity, antibacterial activity, discoloration potential and cytotoxicity of GI, Turmeric and Chlorhexidine mouth rinses.

The null hypotheses tested were : (1) there are no differences in the antibacterial efficacy of 0.2% GI, 0.1% Turmeric and 0.2% CHX mouthrinses; (2) there are no differences in the retention ability of 0.2% GI, 0.1% Turmeric and 0.2% CHX mouthrinses (3) there are no differences in the staining potential of 0.2% GI, 0.1% Turmeric and 0.2% CHX mouthrinses and (4) there are no differences in the cytotoxicity of 0.2% GI, 0.1% Turmeric and 0.2% CHX mouthrinses.

## Methodology

### Ethics

Ethical clearance was acquired from Institutional ethics committee review board for the use of human extracted teeth (IEC 799/2020).

### Sample Size determination

The sample size in the study was based on an *in vitro* assessment of characteristic properties of a guava extract mouthrinse.^24^ G*Power software (Heinrich Heine University, Dusseldorf, Germany) was used to estimate sample size. The sample size was calculated at a confidence interval of 95% and 90% power. The primary variables assessed were substantivity, antimicrobial property and staining property. Considering the effect 0.5, sample size of 16 per group was calculated to assess staining property, antimicrobial property and 18 per group to assess substantivity.

### Preparation of experimental agents

#### Formulation of Garcinia indica (GI) fruit extract Mouth rinse

*Garcinia indica* dried fruit was procured from the university campus and utilized for preparation of the extract. The fruit specimen was identified and authenticated by an authorized botanist. The identified GI extract was validated and provided with a voucher specimen no. *PP627* and was deposited in the department herbarium as part of official protocol. The formulation of the GI fruit extract involved drying the fruit peels and then coarsely powdered. The weighed quantity of the coarse powder was macerated with 70% pure ethanol in the ratio of 1:10 which was stirred occasionally for 7days. Thereafter, it was filtered and the marc was pressed and mixed with the strained liquid. The extract obtained was then concentrated using rota evaporator to remove the solvent residues and a semi-solid mass was obtained. The dried extract was kept in a desiccator till further use. The next step involved determination of Minimal inhibitory concentration (MIC). The MIC was performed on organisms that was commonly found in early supragingival plaque which included *Streptococcus mutans* (ATCC^®^ 25175™), *Streptococcus oralis* (ATCC^®^ 35037™), *Fusobacterium nucleatum* (ATCC^®^ 25586™), *Prevotella intermedia* (ATCC^®^15033™).

Based on the MIC results, a mean value of 2 μg/ml was obtained, which accounted for the preparation of 0.2% *GI* fruit extract mouthrinse. 100 ml of distilled water was used to dissolve 20 mg of GI dried fruit extract, and 0.005% peppermint oil was added to the mixture to enhance flavour and stored at cool environment until further use.

#### Formulation of 0.1% Turmeric mouth rinse

Turmeric extract powder was procured commercially and the mouth rinse was prepared as per the methodology quoted by Waghmare, et al.^17^ (2011). In brief, 100 ml of distilled water was dissolved in 10 mg of curcumin extract. Additionally, 0.005% peppermint oil was added to the mixture to enhance flavour and bring the pH level to 4. The formulated mouthrinse was then stored in a cool environment until further use.

#### Chlorhexidine mouthrinse

0.2% Clohex^®^ Mouth Wash (Periogard-Colgate) was procured commercially.

## Experimental groups

**Figure 1:**
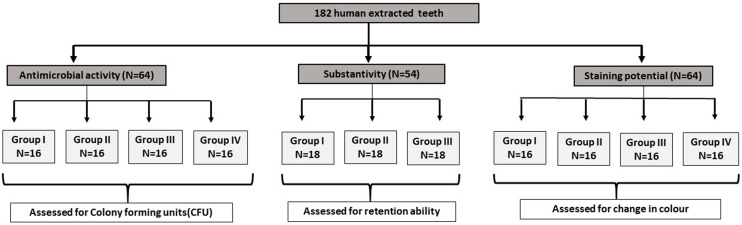
Schematic representation of distribution of samples amongst the experimental groups

The present study included 4 groups. Group I - 0.2% GI fruit extract mouth rinse (Test group), Group II- 0.1% Turmeric mouthrinse (Test group), Group III- 0.2% CHX mouthrinse (Positive control), Group IV – Distilled water (control). A total of 182 tooth sections were used in this experimental study for performing various parameters (i.e. antimicrobial, substantivity and staining properties). ( Figure1 )

## Methodology for assessment of basic properties

### Biofilm development

The methodology for biofilm development was based on the previous study conducted by Guggenheim, et al.^25^ (2001). The microbial biofilm was developed using the following bacterial species; *Streptococcus mitis* (ATCC^®^ 49456™), *Streptococcus mutans* (ATCC^®^ 25175™), *Streptococcus oralis* (ATCC^®^ 35037™), *Fusobacterium nucleatum* (ATCC^®^ 25586™), *Prevotella intermedia* (ATCC^®^15033™).

Sixty four premolars (n=16/ group) extracted for orthodontic purpose from age group of 18-25 years were used. Teeth with caries, resorption and non-carious lesions were excluded. The extracted teeth were thoroughly disinfected and sectioned using a low speed diamond point (Horrico, Berlin, Germany) to form crown fragments, which were shaped into a rectangular design (approximate dimensions 5 mm×2 mm×1 mm). Further, these fragments were placed into 24-well culture plates which had a mixture of artificial saliva, modified fluid universal medium and a buffer solution. The above-mentioned microbial species suspension (200µL) was inoculated anaerobically into the wells at 37°C for 0.5 h, 16.5 h, 40.5 h, and 64.5 h. The medium was replenished by aspirating the used medium from the wells and using fresh medium at the mentioned time intervals.

### Assessment of colony forming units

The crown fragments coated with the biofilm was thereafter cleaned by dipping in 2 ml of physiological saline solution thrice before being immersed for 1 min in 1 ml of the test agents (0.2% CHX, 0.1% Turmeric mouthrinse, 0.2% GI fruit extract mouthrinse, and distilled water). The biofilm was left undisturbed until the final treatment and extracted after 6h by vortexing with 1mL physiological saline. For the 12h time interval, additional aliquots of the grown biofilm were sonified, diluted, and placed on a columbia agar base (Criterion™,Santa Maria, CA, USA ) and incubated anaerobically at 37°C. The EC2™ automatic colony counter (BioMérieux Inc, Hazelwood, MO, USA) was used to count colony-forming units (CFU) at 6h and 12h after plating.

### Assessment of substantivity

The methodology to assess the retentive property of the experimental agent was as per the research work conducted by Freitas, et al.^26^ (2003). For the present study, 54 teeth (n=18 per group) were collected and thoroughly cleaned. The tooth specimens were selected as per inclusion and exclusion criteria mentioned in the section of biofilm development. Further, the tooth specimens were decoronated with a water-cooled diamond saw to obtain the crowns. The crown fragments were then inserted in polystyrene resin (Alfa Chemistry™, NY,USA), after which, this resin coated surfaces was removed and a layer of nail paint was applied and randomly allotted to assess the substantivity by 1 min immersion into the respective test agents (i.e.group 0.2% GI fruit extract based mouthrinse, group 0.1% turmeric mouthrinse) and group 0.2% CHX mouth rinse.The mean release of the experimental mouth rinses were recorded at the end of 360 min.^27^ The specimens were immersed in 1 mL of distilled water and kept in glass tubes. An aliquot was removed from the tubes at specified time intervals, and the same volume was then immediately restored and subjected to UV spectrophotometer analysis at 354nm for 0.2% GI, 425nm for 0.1% turmeric and 260nm for 0.2% chlorhexidine.

### Assessment of staining potential

The staining potential was as per the methodology recommended by Addy et al.^28^ (1995). Briefly, a standard tea solution (Marks and Spencer extra-strong loose tea, Chester, UK) was prepared by boiling 8g of tea leaves in 800mL of water for two minutes. The tea solution was cooled for 30 minutes at 4°C before being filtered to get clear of the tea remnants. The tea solution was then left at room temperature for the duration of the experiment. A total of 64 human intact teeth (n=16 per group) were sectioned mechanically and was allotted into four groups (group 0.2%GI fruit extract based mouthrinse, group 0.1% turmeric mouthrinse, group 0.2% chlorhexidine and group distilled water). The tooth specimens were selected as per inclusion and exclusion criteria mentioned in the section of biofilm development. All the specimens were submerged in artificial saliva for two minutes at 37°C, followed by repeated rinsing with 2 mL of distilled water. Then the tooth samples from each group were submerged for two minutes in the groups containing the experimental agents or distilled water. The specimens were immersed for at least an hour in a normal tea solution after being rinsed four times in a row with 2 mL of water. The whole cycle was repeated six times during the course of 11 h. The specimens were dried with compressed air after the final rinse, which included four washes with 2mL of distilled water. The luminosity was then measured with a CIELAB color meter and visualization was done under the spectrophotometer(X-Lite).

The change in colour was calculated using the following formula:


$$\Delta E^{*}ab={\left[{\left(\Delta L^{*}\right)}^{2}+(\Delta a*{)}^{2}+{\left(\Delta b^{*}\right)}^{2}\right]}^{1/2}$$

Where, “L” stands for change in luminosity, “a” stands for red-green axis, “b” stands for yellow-blue axis according to the CIELAB reading and further:


$$\Delta L^{*}=L^{*}\text{ post }-L^{*}\text{ pre }$$

$$\Delta a^{*}=a^{*}\text{ post }-a^{*}\text{ pre }$$

$$\Delta b^{*}=b^{*}\text{ post }-b^{*}\text{ pre }$$

Based on these readings the overall change in colour was calculated (ΔE), and used for the analysis of staining property

## Assessment of cell viability by MTT assay

The present *in vitro* research utilized Chinese hamster V79 cells. The cells were acquired from the National Centre for Cell Science (Pune, India) and were maintained as per ATCC guidelines. Cell culture was carried out in Dulbecco’s Modified Eagle Medium (DMEM; Gibco, Thermo Fisher Scientific, Waltham, MA, USA) supplemented with 10% foetal bovine serum (Gibco) and a 1xantibiotics/antimycotic mix (Antibiotic-Antimycotic, Gibco). The cultured cells were incubated in a 5% CO2 incubator (Eppendorf Galaxy 170 R, Germany) at 37°C. The cells were grown in T-75 flasks (Biolite, Thermo Fisher Scientific) and maintained at 60–70% confluence and routinely sub-cultured by trypsinisation. The influence of the experimental mouthrinses (0.2% GI, 0.1% turmeric and 0.2% CHX) were tested on V79 Chinese hamster fibroblasts by conducting a 3-(4,5-dimethyl-2-thiazolyl)-2,5-diphenyl-2H-tetrazolium bromide (MTT, Sigma–Aldrich) MTT dye reduction assay. The methodology was adapted from a previous study conducted by Mosmann, et al.^29^ (1983). In brief, the V79 fibroblasts were seeded at a density of 10^5^ cells per well in 96 well plate (Nest Biotechnology Co., Jiangsu, China) and incubated at 37^o^ C in a 5% CO_2_ incubator for 24 hours. The three experimental agents were tested for their ability to alter viability of cells. Cisplatin (Aarjey Healthcare Private Limited, Thane) was used as the positive control and DMEM served as the negative control. Different dilutions(0.05%, 0.1% and 0.2%) of each experimental agents were prepared with DMEM for 15 minutes. Then the test agents were eliminated and cells were treated with 20 µL of MTT dye (M5655, Sigma Chemical, St.Louis, MO, USA) formulated in DMEM (without serum) at a concentration of 5 mg/ml and again incubated at 37°C in a 5% CO_2_ incubator for another 4 h. Following which, the MTT containing medium was discarded and 100 µL of dimethyl sulfoxide was added to all the wells to dissipate the purple-colored formazan crystals that was formed. Using multi-well plate reader (Lonza’s Nebula^®^ Absorbance Reader, Wayne, PA, USA). The amount of formazan formed was directly proportional to the viable cells. The absorbance at 570nm was recorded and the percentage of viable cells was calculated using the following formula:


$$\Delta E^{*}ab={\left[{\left(\Delta L^{*}\right)}^{2}+{\left(\Delta a^{*}\right)}^{2}+{\left(\Delta b^{*}\right)}^{2}\right]}^{1/2}$$

## Statistical analysis

The data collected were entered into a Microsoft Excel spreadsheet and analyzed using IBM SPSS Statistics, Version 22 (Armonk, NY: IBM Corp). Descriptive statistics were presented in the form of mean and standard deviation. Since the data exhibited a normal distribution with Shapiro-Wilk test, parametric tests were employed.

To assess the variation in antimicrobial properties across distinct time intervals, a mixed ANOVA was employed, followed by a Sidak test for *post hoc* analysis. Comparative evaluations of cytotoxicity and staining properties among the study groups were executed using one-way ANOVA, and subsequent Tukey post hoc tests were applied. A significance threshold of P<0.05 was established to denote statistical significance in the study findings.

## Results

### Antibacterial property

At baseline, the mean CFU scores in Group 0.2% GI fruit extract, Group 0.1%turmeric , Group 0.2%CHX and Group distilled water were standardised (p=0.40) [ Table 1 ]. After 6th hr time interval, a substantial decrease in the mean CFU counts were noted from baseline in Group 0.2% GI (16.50±7.08), Group 0.1%turmeric (106.50±29.45), Group 0.2%CHX (18.88±4.96). However, in Group distilled water, an added increase was observed in the mean CFU value (393.56±18.32) which resulted in a statistical significance among the experimental groups (p<0.001). [ Table 1 ]. Further, at the end of 12 hr study time period, a slight elevation in the mean CFU levels was observed in Group 0.2%GI (20.81±4.81), Group 0.2%CHX ( 28.88±5.41) and Group distilled water (433.06±24.10). Interestingly, a decline in the mean microbial counts were noticed in Group 0.1%turmeric (51.88±16.03). The change in the mean CFU among the groups resulted in statistically significant difference at the 12hr time interval (p<0.001) [ Table 1 ]. Pair-wise comparison, at baseline measurement, there was no significant difference between all the four groups tested (P>0.05). At 6 and 12 hr time periods, both Groups 0.2% GI and Group 0.2% CHX demonstrated maximum reduction in CFU’s with no significant difference between them (P>0.05). However, Group 0.1%turmeric had minimal reduction in CFU score when compared to Groups 0.2%GI fruit and Group 0.2%CHX, which exhibited statistical significance (P<0.001). Group distilled water had least reduction in the CFU’s when compared to test agents ( Table 1 ).

**Table 1:** Comparison of antibacterial activity at different time intervals in each study group.

Group	N	Time						F	p-value
		Baseline		After 6 Hrs		After 12 Hrs			
		Mean	SD	Mean	SD	Mean	SD		
0.2%GI	16	372.13	11.95	16.50^a,#^	7.08	20.81^a,€^	4.81	3,021.86	<0.001*
0.1%Turmeric	16	378.5	13.83	106,5	29.45	51.88	16.03	2,267.21	<0.001*
0.2% CHX	16	379.13	9.29	18.88^b,#^	4.96	28.88^b,€^	5.41	3,051.38	<0.001*
Distilled water	16	375.87	15.11	393,56	18.32	433,06	24.1	75.07	<0.001*
F		1		1,589,16		2,877.73			
p-value		0.40(NS)^#^		<0.001*		<0.001*			

Mixed ANOVA,

Within-Subjects Effects – Time - F = 6703.81, p<0.001* Time * Group - F = 1044.81, P<0.001*

Between-Subjects Effects – Group - F = 2297.01, P<0.001*

Pairwise comparison between groups read vertically.

All pair comparison at baseline and those with similar superscript(a,b) are Non-Significant

Pairwise comparison between different time intervals read horizontally.

All pair comparison with similar superscript(#,€) are Non-Significant All other pairwise comparisons are statistically Significant, p<0.05

*p<0.05 Statistically Significant, p>0.05 Non Significant, NS

### Substantivity

The results related to the substantivity are summarized in Table 2 . At the end of 360 min, samples in Group 0.2% GI exhibited a mean desorption of 5.02±3.04, samples in Group 0.1% Turmeric showed a mean desorption of 12.47±5.84 and samples in Group 0.2% CHX presented with a mean desorption of 4.13±2.25 .Pairwise comparison revealed Group 0.1% Turmeric had a higher mean release rate compared to Group 0.2% GI and Group 0.2%CHX (p<0.001). However, there was no statistical difference observed between Groups 0.2% GI and 0.2% CHX. (p>0.05).

**Table 2:** Mean ± SD values of experimental mouth rinses (0.2%GI, 0.1%Turmeric and 0.2%CHX) release rate at 360 min time interval.

Time (mins)	Study groups	N	Mean (μg/ml)	SD	Min	Max	ANOVA	
							F	p-value
360	0.2%GI	18	5.02^a^	3.04	0.2	10.5	23.39	<0.001*
	0.1% Turmeric	18	12.47	5.84	3.7	21.9		
	0.2% CHX	18	4.13^a^	2.25	0	8		

*p<0.05 Statistically Significant, p>0.05 Non Significant, NS

Pairwise comparison between groups with superscript ‘a’ - Non Significant, p>0.05

All other pairwise comparisons are statistically Significant, p<0.05

### Staining property

The mean ΔL, Δa, Δb values for all the groups measured are summarized in Table 3 . All the experimental groups showed a significant difference (p<0.001) in overall colour change (ΔE) except group distilled water ( Table 4 ). The mean DE value were 7.61±2.4 for Group 0.2%GI fruit extract, 7.32±4.9 for Group 0.1%turmeric mouth rinse and 18.65±8.3 for Group 0.2% CHX mouthrinse which displayed the maximum colour change (P<0.001). On pairwise comparison, there was no statistically significant difference seen between Group 0.2%GI fruit extract mouthrinse and Group 0.1% turmeric mouthrinse (p=0.99). However, 0.2% CHX displayed a notable change in overall colour (ΔE) which was statistically significant compared to 0.2% GI and 0.1% Turmeric (p<0.001) ( Table 4 ).

**Table 3:** Comparison of ∆a, ∆b, and ∆L between the study groups for evaluation of staining potential.

	Study groups (mouthrinses)	Mean	SD	Min	Max	ANOVA	
						F	p-value
ΔL	0.2%GI	-3.88	2.24	-8.66	-0.63	38.33	<0.001*
	0.1%turmeric	-4.12	3,.53	-12.29	1.92		
	0.2%chlorhexidine	-16.93	8.57	-32.85	-2.7		
	Distilled water	0	0	0	0		
Δa	0.2%GI	1.72	0.8	0.01	2.76	27.73	<0.001*
	0.1%turmeric	1.79	1.28	0.16	4.71		
	0.2%chlorhexidine	3.86	1.86	1.6	8.06		
	Distilled water	0	0	0	0		
Δb	0.2%GI	-5.79	2.64	-9.98	-0.66	28.6	<0.001*
	0.1%turmeric	-4.96	4.44	-16.28	0.38		
	0.2%chlorhexidine	4.27	4.74	-4.24	11.63		
	Distilled water	0	0	0	0		

ANOVA statistical test.

*p<0.05 Statistically Significant, p>0.05 Non Significant, NS

**Table 4:** Pairwise comparison of overall colour change (∆E) between the study groups.

	(I) Study groups	(J) Study groups	Mean difference	Std. Error	p-value	95% Confidence interval	
	(Mean±SD)	(Mean±SD)	(I-J)			Lower bound	Upper bound
∆E	0.2% GI (7.61±2.42)	0.1% Turmeric (7.32±4.94)	0.29	1.76	0.99(NS)	-4.36	4.95
		0.2% Chlorhexidine (18.65±8.31)	-11.04	1.76	<0.001*	-15.69	-6.38
		Distilled Water (0±0)	7.61	1.76	<0.001*	2.96	12.27
	0.1% Turmeric (7.32±4.94)	0.2% Chlorhexidine (18.65±8.31)	-11.33	1.76	<0.001*	-15.99	-6.68
		Distilled water (0±0)	7.32	1.76	0.001*	2.66	11.97
	0.2% Chlorhexidine (18.65±8.31)	Distilled water (0±0)	18.65	1.76	<0.001*	14	23.31

ANOVA, F=38.13, p<0.001*

Tukey Post Hoc Test

*p<0.05 Statistically Significant, p>0.05 Non Significant, NS

### Cell viability

**Figure 2:**
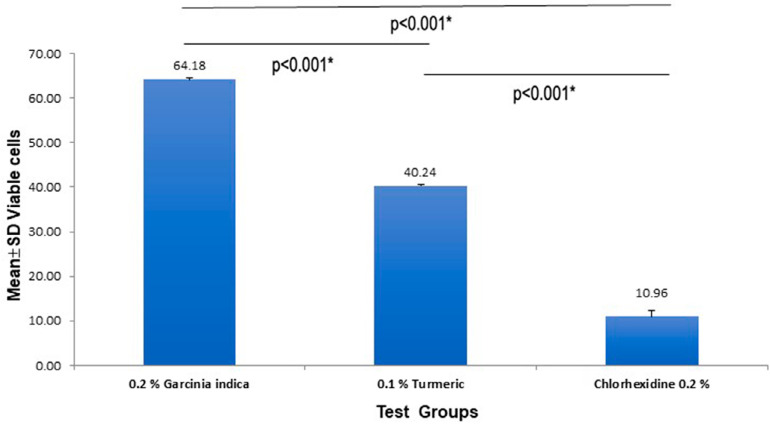
Bar Plot describing mean± SD of viable V79 cells in presence of experimental mouth rinses (0.2%GI, 0.1%Turmeric and 0.2% CHX).

Results of the MTT assay are depicted in Figure 2 .Group 0.2% GI showed the highest number of viable cells (64.17±0.29) compared to Group 0.1% turmeric( 40.24±0.34) and Group 0.2% CHX (10.95±1.40) (p<0.001). When Groups 0.1% turmeric and 0.2% CHX were compared, the latter was found to be more cytotoxic (p<0.001).

## Discussion

In order to evaluate the antibacterial property, a multispecies biofilm comprising of supragingival plaque micro-organisms were developed onto the tooth specimens to mimic the oral environment. The findings observed at the 6^th^ and 12^th^ hour study interval revealed decrease in microbial counts in all the three experimental groups(0.2% GI,0.1%turmeric and 0.2% CHX).This indicates that the tested mouth rinses effectively demonstrated their antibacterial properties. When examining the individual experimental mouth rinses, it was evident that 0.2% CHX and 0.2% GI mouth rinses displayed highest reduction in the CFU counts compared to 0.1% Turmeric mouthrinse . Hence, the first null hypothesis that, there are no differences in the antimicrobial efficacy of 0.2% GI, 0.1% Turmeric and 0.2% CHX mouth rinses has to be rejected. The current research provides evidence that 0.2% GI extract mouthrinse exhibited remarkable antibacterial effectiveness akin to that of 0.2% chlorhexidine mouth rinse. This notable outcome can be attributed to the hydroethanolic formulation of 0.2% GI fruit rind extract which facilitates easier dissolution. Moreover, ethanolic extraction assist in release of active ingredients like anthocyanin, isogarcinol, garcinol and hydroxy citric acid^30 , 31^ which could enhance the quality of the oral rinse. Likewise, other preliminary studies conducted by Sushma et al.^23^ and Nagendra et al^32^ also observed the antimicrobial effectiveness with GI extract. There is high‐quality evidence of anti-plaque and antimicrobial effect with of the use of CHX mouthrinse.^33 , 34^Similar results were observed in the present study, which demonstrated the dominant effect of 0.2% CHX in reducing microbial counts. Various authors have used Chlorhexidine mouth rinse on diverse oral bacteria and derived similar inferences.^35 , 36^ Despite the high efficacy of 0.2% GI and 0.2% CHX in reducing microbial levels from baseline to 12^th^ hr study time period, the 0.1% Turmeric mouth rinse group also exhibited a decline in microbial colonies during the 6^th^ to 12^th^ hr interval. Comparable decline in growth of mature biofilm was documented in a research conducted by Bomdyal, et al.^37^(2017) where the antimicrobial properties of turmeric against periodontopathogens were evident at the end of 48 hours. Contrary to the findings of the present study, Waghmore, et al.^17^ (2011) observed no significant difference in the microbial reduction when 0.1% Turmeric was compared to 0.2% CHX mouthrinse. Discrepancies in results might be due to the variations in microbial strains used, potentially affecting the estimation of colony forming units.

An essential aspect for a mouthrinse is its ability to retain in the oral cavity for an adequate duration, ensuring prolonged therapeutic advantages. The observations of the present study revealed 0.2% CHX and 0.2% GI had maximum retention of mouth rinses on the tooth specimens at the end of 360 min. However, 0.1% Turmeric mouth rinse exhibited higher mean release during the same time period, leading to a significant difference among the tested groups. Hence, the second null hypothesis, that there are no differences in the retention ability of 0.2% GI, 0.1% Turmeric and 0.2% CHX mouth rinses was rejected. Substantivity of the mouth rinses is a basic feature for providing antimicrobial efficacy. Earlier studies performed have stated that the extended action of CHX mouth rinse within the oral cavity can be attributed to its adsorption on the tissue surfaces, resulting in plaque inhibition.^4 , 38^ The current study used 360 min time interval for investigating the substantivity property. The rationale was based on previous studies related to the substantivity of gold standard, CHX mouth rinse.^28 , 39^As per the literature search and author’s knowledge, this is the first study to investigate the substantivity of GI fruit extract and turmeric mouth rinses. Previous research has indicated that single dose of CHX mouth rinse led to detectable microbial reduction for 5h or more.^40^Hence, the retentive potential of both 0.2% GI and 0.1% Turmeric would have contributed to its antimicrobial effect in this study.

Another crucial aspect that was evaluated in this study was the ability of the test mouth rinses to discolour teeth. One of the primary adverse effects of prolonged use of CHX mouth rinse is the discoloration of teeth.^41^ The knowledge that CHX mouth rinses induce external tooth discoloration has been established through various studies.^41 , 42^ In the current study, 0.2%GI and 0.1% turmeric mouth rinses showed an overall colour change on the tested tooth specimens. However, this colour change was comparatively less pronounced with the discoloration observed with 0.2% CHX mouthrinse over the study duration. Thus, the third null hypothesis that, there are no differences in the staining potential of 0.2% GI, 0.1% Turmeric and 0.2% CHX mouth rinses has to be rejected. The presence of anthocyanins^20^ in GI extract and curcuminoids^43^in turmeric may have attributed to the overall colour change. Findings from a previous *in vitro* study exploring factors influencing the development of stains associated with CHX have demonstrated that the frequency of CHX exposure directly and significantly influenced the progression of staining.^44^ In the present study, the experimental cycle was repeated for 6 times over a period of 11 hours, which tried to mimic a clinical situation. Despite the similar methodology followed for 0.2% GI and 0.1% Turmeric mouth rinses, the discoloration effect was less. Hence, this characteristic feature could permit its use has an oral health regimen.

The viability of the oral cells after the use of mouth rinses is a critical factor that needs to be investigated. Our results exhibited GI mouthrinse of 0.2% concentration displayed a highly positive effect on the V79 cell viability compared to 0.1% turmeric mouthrinse. Whereas, 0.2% CHX mouthrinse was found to be largely cytotoxic to the V79 cells. Hence, the fourth null hypothesis that, there are no differences in the cytotoxicity of 0.2% GI, 0.1% Turmeric and 0.2% CHX mouth rinses had to be rejected. Although CHX is known to possess strong antiplaque and antimicrobial effect, it has also been reported to be cytotoxic to human gingival fibroblasts at 0.2% concentration.^45^In a separate investigation, authors observed decreased cell survival and discontinuity in cell migration, even at concentration as minimal as 0.002% for human fibroblasts, osteoblasts and myoblasts. These findings highlight the significant toxic effects of CHX at levels considerably lower than those routinely administered in clinical settings.^46^ Earlier reports comparing the effect of turmeric and CHX on human fibroblast viability and migration have demonstrated, turmeric to have less cytotoxicity compared to CHX at all concentrations and at varying time periods.^47 , 48^

Few distinctive properties required for an effective mouth rinse have been explored and investigated in the current study. However, the present research was conducted *in vitro* , and the results may not be proportional to and translated into clinical effectiveness. This study may have few limitations. Firstly, the antibacterial effectiveness of the mouth rinses was assessed by counting the bacterial colonies (CFU). It is important to note that only viable bacteria capable of multiplying and forming colonies are measured, potentially underestimating the count due to the presence of viable but non-culturable bacteria (VBNC).Further, studies utilizing molecular techniques are necessary for more accurate results on the antimicrobial impact of the tested mouth rinses. Although the MTT assay was used to test the cytotoxicity of the mouth rinses, it only provides a preliminary understanding of short-term toxicity, which may not reflect the time-dependant nature of cytotoxicity.^49^Hence, long term toxicity of these mouth rinses is necessary for comprehensive results. Also, the cultured V79 cells used in this study represent cell response observed in isolation and may not symbolize the complete tissue reaction in clinical practice, which is influenced by multiple factors inclusive of rinse concentration, volume, tissue histology, exposure duration and individual susceptibility. Thus, the observed cell damage *in vitro* may be less pronounced in clinical settings due to the host detoxification mechanisms. Hence, further *in-vivo* clinical trials are required to learn the usefulness of these experimental agents as an alternative to CHX mouthrinse.

## Conclusion

This study supports 0.2% *Garcinia indica* fruit extract mouthrinse and 0.1% turmeric mouthrinse as potential natural alternatives to Chlorhexidine mouthrinse. They offer antibacterial efficacy, retention ability, less staining potential and minimum cytotoxicity. These findings highlight the viability of these natural alternatives in oral health care.
